# GA-SVR Optimized Surface-Enhanced Raman Spectroscopy for Rapid Detection of Ciprofloxacin Residues in Chicken Blood

**DOI:** 10.3390/bios16050259

**Published:** 2026-05-01

**Authors:** Gaoliang Zhang, Zihan Ma, Chao Yang, Yang Liu, Tianyan You, Jinhui Zhao

**Affiliations:** 1 Laboratory of Modern Agricultural Equipment and Technology, Ministry of Education, School of Agricultural Engineering, Jiangsu University, Zhenjiang 212013, China; gaoliangzhang2025@163.com; 2 Jiangxi Key Laboratory of Modern Agricultural Equipment, Jiangxi Agricultural University, Nanchang 330045, China; 13518632789@163.com (Z.M.); 13204921315@163.com (C.Y.); 3 College of Science and Engineering, James Cook University, Townsville, QLD 4811, Australia; 4 College of Agricultural Equipment Engineering, Henan University of Science and Technology, Luoyang 471003, China

**Keywords:** ciprofloxacin, chicken blood, surface-enhanced Raman spectroscopy (SERS), gold colloid, genetic algorithm (GA), support vector regression (SVR)

## Abstract

Ciprofloxacin residues in chicken blood pose a potential food safety risk; however, rapid detection methods for complex chicken blood matrices are lacking. This study aimed to establish a surface-enhanced Raman spectroscopy (SERS) method for the rapid detection of ciprofloxacin in chicken blood using gold colloid as the SERS substrate. Gold colloid was synthesized via the Frens method with slight modification, and key SERS detection conditions were systematically optimized to maximize SERS intensities at 1265 cm^−1^, including the amount of trisodium citrate solution, the electrolyte type, the amount of gold colloid, the amount of NaCl solution, and the adsorption time. Raw SERS spectra were pretreated with adaptive iteratively reweighted penalized least squares (air-PLS) combined with Savitzky–Golay (SG) smoothing. A genetic algorithm (GA) was used to extract characteristic Raman shifts, and a GA-SVR prediction model with radial basis function (RBF) as the kernel was constructed, with its performance compared with multivariate linear regression (MLR) and partial least squares regression (PLSR) models. The GA-SVR model exhibited the best performance, with a coefficient of determination for the calibration set ($R_{c}^{2}$) value of 0.9893 and for the prediction set ($R_{p}^{2}$) value of 0.9874. The root mean square error of calibration (RMSEC) and prediction (RMSEP) were 1.2953 and 1.8617, respectively, outperforming the MLR and PLSR models. These results demonstrate that the SERS method combined with GA-SVR enables rapid quantitative detection of ciprofloxacin residues in chicken blood, providing a technical reference for monitoring veterinary drug residues in livestock and poultry products.

## 1. Introduction

Ciprofloxacin, a third-generation quinolone antibiotic, has the advantages of a broad antibacterial spectrum, strong antibacterial activity and low price, and is widely used in the prevention and treatment of bacterial infections in poultry breeding [1]. However, its irrational use and abuse can lead to residues in animal-derived foods such as chicken meat, chicken blood and eggs [2]. Long-term consumption of food contaminated with ciprofloxacin residues not only causes gastrointestinal discomfort, allergic reactions, and neurotoxicity in humans but also induces the emergence of drug-resistant bacteria, seriously endangering human health and public health security [3,4]. Unlike chicken meat that can only be detected after slaughter and eggs, live chicken blood is an easily collectible target for non-destructive and reproducible in vivo monitoring, enabling real-time continuous detection of ciprofloxacin in live chickens. The rapid quantification of ciprofloxacin residues in chicken blood can timely determine the slaughter qualification of chickens, providing key technical support for pre-slaughter screening of chickens. Currently, the common detection methods for ciprofloxacin residues include high-performance liquid chromatography (HPLC) [5], liquid chromatography-tandem mass spectrometry (LC-MS/MS) [6], and enzyme-linked immunosorbent assay (ELISA) [7]. HPLC and LC-MS/MS have the advantages of high detection accuracy and good separation effect, but they also have the disadvantages of complex sample pretreatment, long detection time, expensive instruments and professional operation requirements, which are not suitable for on-site rapid detection [8]. Although ELISA has the characteristics of simple operation, high throughput and low cost, its detection sensitivity and specificity are relatively low, and it is easy to produce false positive results [9]. Therefore, establishing a novel, rapid and cost-effective method for detecting ciprofloxacin residues in chicken blood that overcomes the limitations of conventional methods is of great significance for ensuring food safety and safeguarding human health.

Surface-enhanced Raman spectroscopy (SERS) is a novel spectral analysis technique derived from traditional Raman spectroscopy. It can significantly enhance the Raman signals of target molecules adsorbed on noble metal nanostructures (e.g., gold, silver and copper) and features the advantages of rapid detection, high sensitivity and simple sample pretreatment [10]. As a typical SERS substrate, gold colloid is easy to prepare, shows good biocompatibility, strong SERS enhancement and stable performance, and has been widely used in the detection of antibiotic residues [10,11]. The SERS detection conditions (e.g., the applied amount of reducing agent and electrolyte type) have a significant impact on the SERS enhancement effect. Therefore, optimizing the detection conditions is key to improving the SERS detection sensitivity of ciprofloxacin residues in chicken blood.

In the quantitative detection by SERS, the original spectral data contains abundant redundant information and noise, which can adversely affect the accuracy and stability of the quantitative model [12]. Spectral pretreatment can effectively eliminate the interference caused by noise, extract effective spectral characteristics, and improve the performance of the quantitative model [13]. In addition, feature extraction technology (such as principal component analysis, partial least squares, and genetic algorithm) can screen out the characteristic variables closely related to the target substance from the high-dimensional spectral data, reduce the complexity of the model and avoid overfitting [14]. Genetic algorithm (GA) is a global optimization algorithm based on biological evolution theory, characterized by strong global search capability, good robustness to noise and interference, and easy implementation with simple parameter tuning, which has been widely used in spectral feature extraction [15]. Support vector regression (SVR) is a machine learning method based on statistical learning theory, characterized by strong generalization ability and excellent fitting performance for small-sample, high-dimensional, and nonlinear spectral data, which has been widely used in quantitative SERS analysis [16]. Combining GA with SVR (GA-SVR) can not only screen out effective characteristic variables through GA to reduce data dimensionality and model complexity but also achieve the nonlinear fitting between spectral data and target substance content through SVR, which can effectively improve the accuracy and stability of the quantitative model [14]. However, most existing studies have focused primarily on the detection of ciprofloxacin in chicken muscle and water solution samples, including drinking water and breeding water, and still present certain limitations, including inadequate optimization of detection conditions, insufficient anti-interference capability in complex matrices, and relatively low accuracy and robustness of the established quantitative models [14,15,16]. Compared with the above samples, chicken blood is a more challenging matrix for analysis because it contains abundant biomolecules, especially hemoglobin, which can lead to severe competitive adsorption on SERS substrates and attenuation of characteristic signals during spectral collection. It is also a food safety monitoring target, and its residual level directly reflects the ciprofloxacin metabolism status of live chickens. Notably, the application of SERS combined with chemometrics for ciprofloxacin detection in chicken blood has not been reported, and there is a lack of optimization of detection conditions and multivariate analysis for SERS detection of ciprofloxacin in chicken blood.

In this study, gold colloid was synthesized and used as the SERS substrate, and the SERS detection conditions for ciprofloxacin in chicken blood were systematically optimized. Different spectral pretreatment methods were compared to select the optimal pretreatment protocol. GA was employed to extract SERS characteristic variables, and a GA-SVR quantitative prediction model was established. The overall experimental workflow of this study is shown in Figure S1. The novelty of this study lies in the combination of SERS condition optimization with the GA-SVR algorithm, and this method was first applied to the rapid detection of ciprofloxacin in chicken blood. The performance of the GA-SVR model was compared with that of multivariate linear regression (MLR) and partial least squares regression (PLSR) models, thereby providing a new method for the rapid and accurate detection of ciprofloxacin residues in chicken blood.

## 2. Materials and Methods

### 2.1. Reagents and Materials

Chicken whole blood was purchased from the local market near Jiangxi Agricultural University. An appropriate amount of chicken blood samples was sent to a professional testing institution for detection, and the test report showed that no ciprofloxacin was detected in the samples.

All reagents were of analytical grade unless otherwise specified. Ciprofloxacin (≥98%, Shanghai Yuanye Biotechnology Co., Ltd., Shanghai, China), food-grade sodium citrate (about 99%, Shandong Ensign Industrial Co., Ltd., Weifang, China), chloroauric acid trihydrate (≥49.0%, Sigma-Aldrich Trading Co., Ltd., Shanghai, China) and formic acid solution (98%, Shandong Keyuan Biochemical Co., Ltd., Heze, China) were used in this study. Anhydrous magnesium sulfate (MgSO_4_, ≥98%), sodium chloride (NaCl, ≥99.5%), calcium chloride (CaCl_2_, ≥99.5%), potassium chloride (KCl, 99.5%), and acetonitrile solution (≥99.5%) were purchased from Xilong Scientific Co., Ltd., Shantou, China.

### 2.2. Instruments and Equipment

The experimental instruments used in this study were as follows: a portable Raman spectrum acquisition system, which was mainly composed of a QE65 Pro Raman spectrometer (Ocean Insight, Orlando, FL, USA), a 785 nm laser (Ocean Insight, USA), an optical fiber, a Raman spectrum sampling accessory, and a computer; a thermostatic water bath (WB100-4F, JOANLAB Equipment Co., Ltd., Huzhou, China); an electronic balance (FA2004, Shanghai Sunny Hengping Scientific Instrument Co., Ltd., Shanghai, China); an intelligent thermostatic magnetic stirrer (ZNCL-T, Gongyi Chuanyuan Instrument Manufacturing Co., Ltd., Gongyi, China); a numerical control ultrasonic cleaner (KQ-500DE, Kunshan Ultrasonic Instrument Co., Ltd., Kunshan, China); a high-speed centrifuge (PK-165, Hunan Pingke Scientific Instrument Co., Ltd., Changsha, China); and a vortex mixer (VORTEX-6, Haimen Kylin-Bell Lab Instruments Co., Ltd., Haimen, China).

### 2.3. Preparation of Standard Stock Solutions

The 0.1% formic acid–acetonitrile solution was prepared as follows: 1 mL of formic acid solution was accurately pipetted into a 1000 mL brown volumetric flask, followed by diluting to the marked volume (1000 mL) with acetonitrile solution, and the mixture was vortexed thoroughly until homogeneous.

The ciprofloxacin standard stock solution was prepared by the following procedure: 5 mg of ciprofloxacin standard substance was precisely weighed and transferred into a brown volumetric flask. It was dissolved in 100 mL of ultrapure water with ultrasonic assistance, and then, a ciprofloxacin standard stock solution with a concentration of 50 mg/L was obtained. During the experiment, this stock solution was serially diluted with ultrapure water to prepare ciprofloxacin working solutions of different concentrations as required.

### 2.4. Preparation of Chicken Blood Samples

First, food-grade sodium citrate solution (10 g/L) was added as an anticoagulant into a 100 mL centrifuge tube, and chicken blood was added at a volume ratio of anticoagulant to chicken blood of 1:3, followed by storage at low temperature for later use. Then, 9 mL of the above-treated chicken blood was pipetted into a 50 mL centrifuge tube, and 1 mL of ciprofloxacin standard solutions with different concentrations was added; the mixture was vortexed for 2 min and ultrasonically oscillated for 5 min to obtain chicken blood samples containing different concentrations of ciprofloxacin. Subsequently, 2 mL of the prepared chicken blood sample was transferred into a 5 mL microcentrifuge tube, and 2 mL of 0.1% formic acid–acetonitrile solution was added and mixed thoroughly. After vortexing for 30 s and ultrasonically oscillating for 10 min, the microcentrifuge tube was placed in a freezer. After 5 min, the sample was taken out of the freezer and centrifuged at 14,000 r/min for 10 min using a high-speed centrifuge, and the supernatant was collected for subsequent detection.

### 2.5. Preparation of Gold Colloid Substrate

Gold colloids used in this study were synthesized based on the Frens method [17] with slight modifications, and the specific procedures were as follows: First, 100 mL of 1 mM HAuCl_4_ solution was added to a round-bottom flask. After the solution was heated to boiling, 3.3 mL of 1% trisodium citrate solution was immediately added, and the mixture was continuously stirred and heated for 30 min. Subsequently, the obtained colloidal solution was cooled to room temperature, and the gold colloids adopted in this study were finally obtained.

### 2.6. Collection of Raman Spectra for Sample

A specific volume of gold colloid, 20 μL of the sample to be tested, and a specific volume of electrolyte solution were sequentially added to a quartz vial. After thorough mixing, the mixture was placed into the Raman spectrum sampling accessory, and the Raman spectrum of the sample was collected after adsorption for a certain period of time.

The parameters of the portable Raman spectrum acquisition system were set as follows: integration time of 10 s, laser energy of 800 mW, average scanning times of 2, smoothness of 1, and spectral scanning range of 150–2100 cm^−1^. For subsequent data analysis, the Raman spectral range of 400–1800 cm^−1^ was selected.

### 2.7. Feasibility Test Scheme of SERS Detection

To evaluate and comparatively analyze the feasibility of SERS detection for ciprofloxacin in chicken blood, the SERS spectra of four groups of samples were collected, including: gold colloid + NaCl solution, gold colloid + ciprofloxacin aqueous solution (10 mg/L) + NaCl solution, gold colloid + blank (ciprofloxacin-free) chicken blood extract + NaCl solution, and gold colloid + chicken blood extract containing ciprofloxacin+ NaCl solution.

### 2.8. Optimization Test Scheme of SERS Detection Conditions

A single-factor optimization method was adopted to investigate the effects of five key factors on the SERS intensity of ciprofloxacin in chicken blood samples at 1265 cm^−1^, including the amount of trisodium citrate solution for gold colloid synthesis, electrolyte type, gold colloid applied amount, NaCl solution applied amount and adsorption time. Five parallel samples were set for each experimental condition.

To explore the effect of gold colloids prepared with different applied amounts of trisodium citrate on the SERS intensity of ciprofloxacin in chicken blood samples, gold colloids were first prepared under seven conditions with the addition of 1% trisodium citrate solution at different volumes (2.9, 3.1, 3.3, 3.5, 3.7, 3.9 and 4.1 mL). Then, 500 μL of gold colloid, 20 μL of chicken blood extract containing ciprofloxacin and 50 μL of 0.1 mol/L NaCl solution were added into a quartz bottle, mixed thoroughly, and the SERS spectrum was collected after adsorption for 0 min. The characterizations of seven gold colloids were analyzed with transmission electron microscopy (TEM; Hitachi HT7700, Tokyo, Japan) at an operating voltage of 100 kV. Their average particle sizes were calculated based on TEM images of 50 particles. Their absorption spectra were obtained with a Shimadzu UV-1280 UV-vis spectrophotometer (Shimadzu, Tokyo, Japan).

To explore the effect of different electrolytes on the SERS intensity of ciprofloxacin in chicken blood samples, 500 μL of gold colloid, 20 μL of chicken blood extract containing ciprofloxacin and 50 μL of different types of 0.1 mol/L electrolyte solutions (CaCl_2_ solution, MgSO_4_ solution, KCl solution, and NaCl solution) or an equal volume of ultrapure water were added into a quartz bottle, mixed thoroughly, and the SERS spectrum was collected after adsorption for 0 min.

To explore the effect of different gold colloid applied amounts on the SERS intensity of ciprofloxacin in chicken blood samples, different volumes (400, 450, 500, 550 and 600 μL) of gold colloid, 20 μL of chicken blood extract containing ciprofloxacin and 50 μL of 0.1 mol/L NaCl solution were added into a quartz bottle, mixed thoroughly, and the SERS spectrum was collected with an adsorption time of 0 min.

To explore the effect of different NaCl solution applied amounts on the SERS intensity of ciprofloxacin in chicken blood samples, 500 μL of gold colloid, 20 μL of chicken blood extract containing ciprofloxacin and different volumes (40, 45, 50, 55 and 60 μL) of 0.1 mol/L NaCl solution were added into a quartz bottle, mixed thoroughly, and the SERS spectrum was collected with an adsorption time of 0 min.

To explore the effect of different adsorption times on the SERS intensity of ciprofloxacin in chicken blood samples, 500 μL of gold colloid, 20 μL of chicken blood extract containing ciprofloxacin and 50 μL of 0.1 mol/L NaCl solution were added into a quartz bottle, mixed thoroughly, and the SERS spectra were collected at different adsorption times (0, 1, 2, 3, 4 and 5 min).

### 2.9. SERS Quantitative Detection Test Scheme

To establish a SERS quantitative prediction model for the detection of ciprofloxacin in chicken blood samples, 120 chicken blood samples containing ciprofloxacin with concentrations ranging from 1 to 40 mg/L were first prepared. Among these samples, 90 were randomly selected as the training set for model establishment, while the remaining 30 samples served as the prediction set. Subsequently, under the optimized detection conditions, the SERS spectral data in the range of 400–1800 cm^−1^ were selected for the establishment of the SERS quantitative prediction model.

### 2.10. Data Analysis Scheme

To eliminate the aforementioned interference, extract the SERS spectral characteristics truly associated with the ciprofloxacin concentration in chicken blood, and enhance the accuracy of the subsequent prediction model; preprocessing of the original SERS spectra is essential. Based on the general principles of spectral analysis preprocessing and the inherent characteristics of SERS technology, this study systematically investigated the model prediction performance under various spectral preprocessing methods and their combinations. These combinations included adaptive iteratively reweighted penalized least squares (air-PLS) alone, air-PLS combined with Savitzky–Golay (SG) smoothing, air-PLS combined with the first derivative, air-PLS combined with the second derivative, air-PLS combined with normalization, air-PLS combined with standard normal variate (SNV), and air-PLS combined with multiplicative scatter correction (MSC). The air-PLS algorithm was implemented using Matlab R2010b software, while all other spectral preprocessing algorithms were executed in Unscrambler X 10.4 software.

To screen out the characteristic variables that are most sensitive to ciprofloxacin (the target analyte) in chicken blood and capable of resisting matrix interference from the full spectral data, thereby constructing a more robust and universal quantitative analysis model, GA was adopted to select the characteristic Raman shifts of SERS in this study. The Matlab GA-PLS toolbox was employed to screen the effective SERS characteristic Raman shifts for the detection of ciprofloxacin in chicken blood. The relevant parameters of GA were set as follows: the initial population size was 30, the mutation probability was 0.01, the crossover probability was 0.5, the fitness function of GA was constructed based on the root mean square error of cross-validation (RMSECV) value, and the iteration was terminated when the number of iterations reached 150.

On the basis of characteristic Raman shift selection via GA, SVR was utilized to establish a prediction model for the ciprofloxacin content in chicken blood in this study. The Unscrambler software was used to establish the prediction model for ciprofloxacin content in chicken blood. The relevant parameters of SVR were set as follows: the SVR function type was epsilon SVR, the kernel function was the radial basis function (RBF), and the penalty parameters C and Gamma were optimized through grid search. Meanwhile, the prediction performances of GA-SVR models under four different kernel functions (RBF function, sigmoid function, linear function, and polynomial function) were compared.

In addition, two other prediction models, i.e., MLR and PLSR, were established in this study to objectively evaluate the prediction performance of the GA-SVR model.

## 3. Results

### 3.1. SERS Analysis of Ciprofloxacin in Chicken Blood

**Figure 1:**
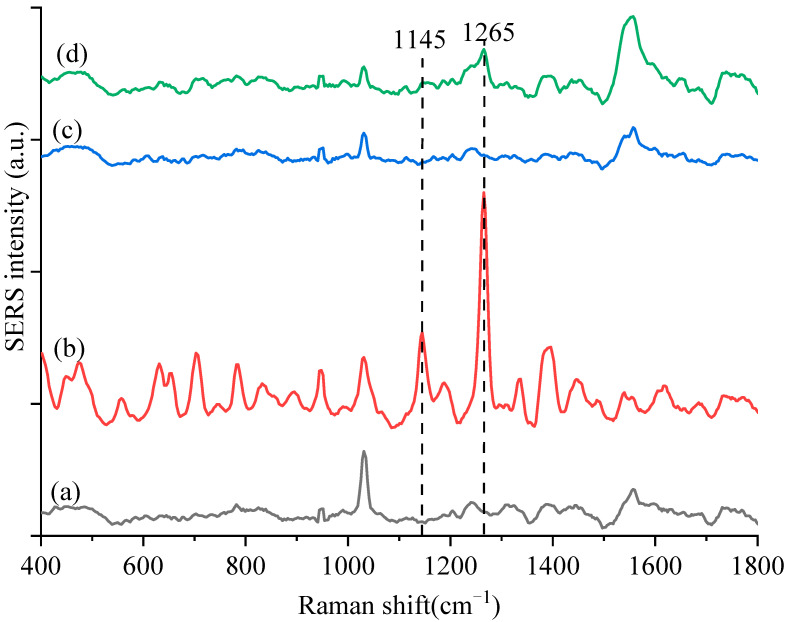
Comparison of SERS spectra of gold colloid in different systems: (a) gold colloid + NaCl solution; (b) gold colloid + ciprofloxacin solution + NaCl solution; (c) gold colloid + chicken blood extraction without ciprofloxacin + NaCl solution; and (d) gold colloid + chicken blood extraction containing ciprofloxacin + NaCl solution.

The SERS spectra of four samples shown in Figure 1 were compared to evaluate the capability of the proposed SERS method for detecting ciprofloxacin residues in chicken blood. It can be seen from Figure 1 that sharp and strong characteristic peaks appeared at 1145 and 1265 cm^−1^ in the SERS spectrum of the ciprofloxacin aqueous solution; no obvious Raman signals were generated at these two positions in the SERS spectrum of the blank chicken blood extract (without ciprofloxacin), which indicates that the chicken blood extract itself did not produce obvious interference signals at these positions, thereby verifying the specificity of the characteristic peaks. This further demonstrates the excellent selectivity of the characteristic peaks at 1145 and 1265 cm^−1^ for ciprofloxacin detection, as the major matrix components in chicken blood have no obvious overlapping Raman signals at these two characteristic peaks, avoiding the main matrix interference of spectral overlap. The characteristic peaks reappeared at these two positions in the SERS spectrum of the chicken blood extract containing ciprofloxacin; although their intensity may be weakened due to the matrix effect, this also confirmed that ciprofloxacin can be detected in the complex chicken blood matrix.

In addition, when comparing the two characteristic peaks at 1145 and 1265 cm^−1^, it was found that the peak at 1265 cm^−1^ in the SERS spectrum of the ciprofloxacin aqueous solution is sharp, with prominent intensity and a high signal-to-noise ratio. More importantly, in the SERS spectrum of the chicken blood extract containing ciprofloxacin, despite the obvious matrix inhibition effect, the characteristic peak at 1265 cm^−1^ can still be clearly identified and maintains good peak shape integrity. Compared to the characteristic peak at 1145 cm^−1^, the characteristic peak at 1265 cm^−1^ is more capable of resisting the effect of the chicken blood matrix. The gold colloid matrix has a specific adsorption affinity for ciprofloxacin in chicken blood, which is superior to the competitive adsorption of chicken blood matrix components.

### 3.2. SERS Optimization of the Detection Conditions for Ciprofloxacin in Chicken Blood

**Figure 2:**
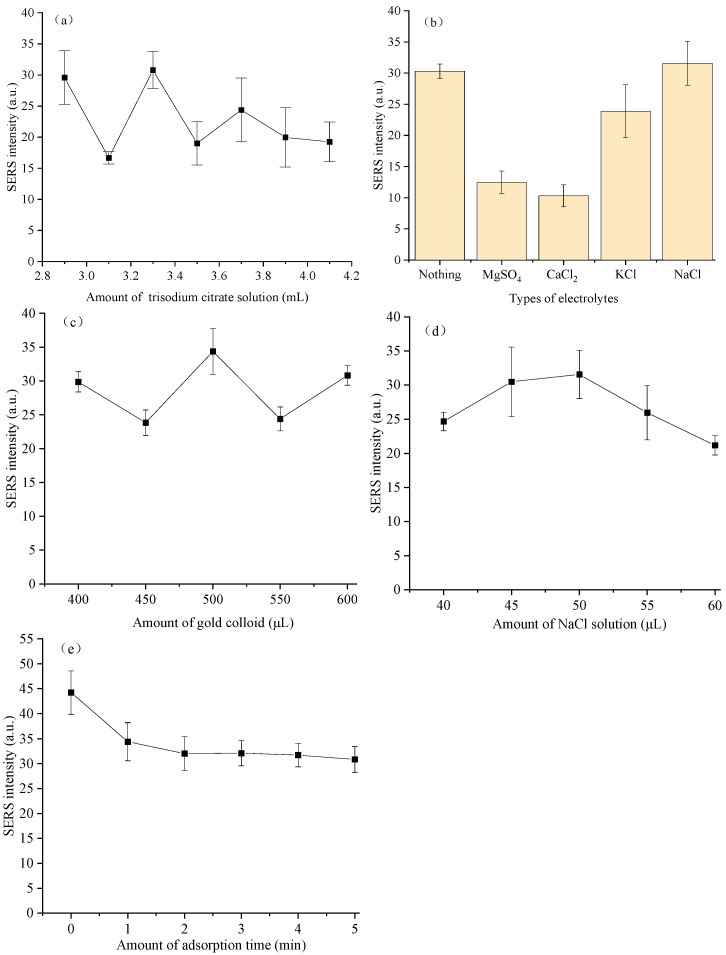
Effects of detection conditions for ciprofloxacin in chicken blood on SERS intensity: (**a**) amount of trisodium citrate solution; (**b**) types of electrolytes; (**c**) amount of gold colloid; (**d**) amount of NaCl solution; and (**e**) adsorption time.

It can be seen from Figure 2a that the SERS signal intensity of ciprofloxacin in chicken blood at the characteristic peak of 1265 cm^−1^ showed significant fluctuations in the gold colloid systems prepared with different applied amounts of trisodium citrate solution (2.9, 3.1, 3.3, 3.5, 3.7, 3.9 and 4.1 mL). As the applied amounts of trisodium citrate solution were increased from 2.9 mL to 3.1 mL, 3.3 mL, 3.5 mL, 3.7 mL, 3.9 mL and 4.1 mL, the average diameters (n = 50) of seven gold colloids synthesized in our lab were decreased from 76 ± 7 nm to 62 ± 7 nm, 63 ± 8 nm, 58 ± 8 nm, 51 ± 8 nm, 39 ± 7 nm, 37 ± 7 nm based on the TEM images (Figure S2). Their UV-vis spectra are shown in Figure S3, and their surface plasmon resonance (SPR) peaks were observed between 535 nm and 548 nm. When the applied amount of trisodium citrate solution was 2.9 mL, the SERS intensity was about 29 a.u.; when the applied amount increased to 3.1 mL, the signal intensity decreased sharply; and when the applied amount of trisodium citrate solution was 3.3 mL, the SERS intensity at the characteristic peak of 1265 cm^−1^ reached its peak value. The test results showed that the gold colloid prepared with an applied amount of 3.3 mL trisodium citrate solution exhibited the optimal SERS enhancement effect on ciprofloxacin in chicken blood.

To explore the effect of electrolyte type on the SERS detection performance of ciprofloxacin in chicken blood, the SERS signal intensity at the characteristic peak of ciprofloxacin at 1265 cm^−1^ was used as the evaluation index to investigate the effects of ultrapure water (no electrolyte) and four common monovalent or divalent electrolyte solutions (i.e., MgSO_4_ solution, CaCl_2_ solution, KCl solution, and NaCl solution) on the SERS intensity of ciprofloxacin in chicken blood, and the results are shown in Figure 2b. Specifically, compared with the blank control group without electrolyte, MgSO_4_ solution, CaCl_2_ solution, and KCl solution reduced the SERS characteristic peak intensity of ciprofloxacin in chicken blood, among which the system with CaCl_2_ solution had the largest reduction range, followed by the system with MgSO_4_ solution, and the system with KCl solution had a slight reduction; the system with NaCl solution showed an enhancement effect, with its intensity higher than that of the other electrolyte groups and the non-electrolyte group. Based on this, NaCl solution was selected as the electrolyte solution for SERS detection of ciprofloxacin in chicken blood in this study.

As the active substrate for SERS detection of ciprofloxacin in chicken blood, the applied amount of gold colloid directly determines the number and distribution of surface-enhanced hot spots in the system, and thus affects the SERS signal intensity of ciprofloxacin in chicken blood. The effect of gold colloid applied amount in the range of 400 to 600 μL on the SERS intensity of ciprofloxacin in chicken blood was investigated, and the results shown in Figure 2c indicated that the SERS intensity exhibited a trend of first decreasing, then increasing, then decreasing, and then rising with the increase in gold colloid applied amount, and that the SERS intensity at the characteristic peak of 1265 cm^−1^ reached its peak value when the gold colloid applied amount was 500 μL. In general, the gold colloid applied amount of 500 μL exhibited the optimal SERS enhancement effect on ciprofloxacin in chicken blood, and either too high or too low an applied amount would lead to a decrease in signal intensity. Based on this, 500 μL was selected as the optimal gold colloid applied amount for SERS detection of ciprofloxacin in chicken blood in this study.

As an electrolyte solution, NaCl solution can induce controllable agglomeration of gold nanoparticles through electrostatic action to construct more SERS hot spots, and its applied amount is one of the key parameters to regulate the SERS enhancement effect. It can be seen from Figure 2d that different applied amounts of NaCl solution had an obvious nonlinear effect on the SERS characteristic signal intensity of ciprofloxacin in chicken blood, and there existed an optimal NaCl solution applied amount. Specifically, when the applied amount of NaCl solution increased from 40 μL to 50 μL, the SERS characteristic peak signal intensity of ciprofloxacin in chicken blood increased accordingly and reached the maximum value at 50 μL. When the applied amount of NaCl solution continued to increase to 60 μL, the SERS signal intensity showed a downward trend. The results showed that the applied amount of 50 μL NaCl solution exhibited the optimal SERS enhancement effect on ciprofloxacin in chicken blood, and any deviation from this applied amount would lead to the attenuation of the SERS signal intensity at the characteristic peak of 1265 cm^−1^.

Adsorption time is an important factor affecting the detection sensitivity of ciprofloxacin in chicken blood by SERS. This study explored the effect of adsorption time in the range of 0 to 5 min on the SERS intensity of ciprofloxacin in chicken blood, and it can be seen from Figure 2e that the SERS intensity was highest at an adsorption time of 0 min. With the extension of adsorption time to 1 min, the SERS intensity decreased rapidly. The downward trend slowed down after 1 min, and the SERS intensity tended to be stable at 2 to 5 min.

### 3.3. Spectral Preprocessing for Ciprofloxacin Detection in Chicken Blood

To establish a high-performance quantitative detection model for ciprofloxacin in chicken blood, prediction models based on GA-SVR were developed under seven different spectral preprocessing methods in this study, and the predictive performance of these methods was systematically compared. Comparison of their spectral preprocessing effects for ciprofloxacin detection in chicken blood is shown in Figure S4. A one-way analysis of variance was conducted to verify the statistical significance of the influence of seven spectral preprocessing methods on model performance, and the results showed a significant difference in the prediction accuracy of GA-SVR models under different preprocessing strategies (*p* < 0.05), which confirmed that seven spectral preprocessing methods exerted a statistically significant influence on the performance of the GA-SVR model. The coefficient of determination for calibration ($R_{c}^{2}$) and coefficient of determination for prediction ($R_{p}^{2}$) of all models were above 0.98 and 0.91, respectively.

As shown in Table 1, the spectral preprocessing method exerted a significant influence on the performance of the GA-SVR model. When air-PLS was used alone, the GA-SVR model already exhibited favorable basic performance ($R_{c}^{2}$ value of 0.9890, $R_{p}^{2}$ value of 0.9823). Although this method could weaken or eliminate background interference such as fluorescence to a certain extent, the root mean square error of prediction (RMSEP) value reached 2.1567, indicating that the generalization ability of the model could be further improved. When combined with SG smoothing, both $R_{c}^{2}$ value (0.9893) and $R_{p}^{2}$ value (0.9874) of the GA-SVR model were increased, and the RMSEP value (1.8617) was lower than that obtained with air-PLS alone.

### 3.4. Results of GA Feature Extraction for Ciprofloxacin Detection in Chicken Blood

**Figure 3:**
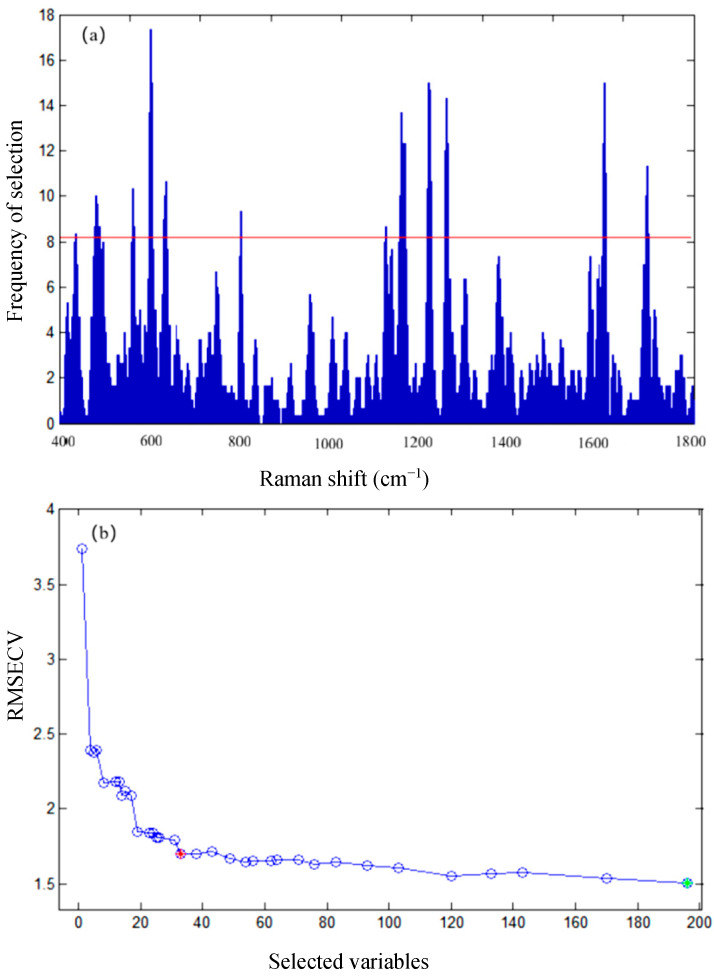
Histogram of frequency of Raman shift selection (**a**) and relationship between the number of selected variables and RMSECV (**b**). In (**a**), the red horizontal line represents the variable selection frequency threshold. In (**b**), the red dot indicates the suggested variable numbers according to the F criterion.

To eliminate redundant variables from the SERS spectra of chicken blood samples, reduce model complexity, and improve quantitative detection performance, GA combined with the F-test criterion was employed for spectral feature extraction in this study. As presented in Figure 3a, the histogram of Raman shift selection frequencies is shown, with the red horizontal line representing the variable selection threshold. This figure illustrates the distribution of Raman shifts selected during multiple GA runs. A total of 33 Raman shift variables were selected, and their distribution was highly uneven, showing several distinct sharp peaks. These peaks indicate that the Raman shifts repeatedly selected by the GA algorithm are critical for establishing the prediction model of ciprofloxacin in chicken blood.

As displayed in Figure 3b, the relationship between the number of selected variables and the RMSECV is presented. With an increase in the number of selected variables, RMSECV decreased rapidly at first and then stabilized.

### 3.5. Results of Quantitative Prediction Model for Ciprofloxacin Detection in Chicken Blood

To optimize the predictive performance of the GA-SVR model for ciprofloxacin detection in chicken blood, the model performance using four different kernel functions, i.e., RBF, sigmoid, linear, and polynomial, was compared in this study. According to Table 2, significant differences were observed in the performance of the GA-SVR models with different kernel functions. The model with the RBF kernel function achieved the best performance, with a $R_{c}^{2}$ value of 0.9893, root mean square error of calibration (RMSEC) value of 1.2953, $R_{p}^{2}$ value of 0.9874, and RMSEP value of 1.8617. The model with the sigmoid kernel function ranked second, while the linear and polynomial kernel functions yielded relatively weak performance. In particular, the polynomial kernel function resulted in an R_p_^2^ value of only 0.9697 and an RMSEP value as high as 2.3295.

To further verify the advantages of the GA-SVR model, the predictive performances of three models—MLR, PLSR, and GA-SVR—were compared in this study. As indicated in Table 3, there were significant differences in the performance of the three models. Among them, the GA-SVR model exhibited the optimal performance, followed by the MLR model, while the PLSR model showed relatively weaker performance.

## 4. Discussion

### 4.1. SERS Detection Condition Optimization Analysis for Ciprofloxacin in Chicken Blood

Trisodium citrate solution acts as both a reducing agent and a stabilizer in the synthesis of gold colloids, and its applied amount indirectly affects the SERS enhancement efficiency of ciprofloxacin in chicken blood by regulating the particle size, morphology, distribution, dispersibility, and local surface plasmon resonance (LSPR) effect of gold nanoparticles in the gold colloid [18]. When the applied amount of trisodium citrate solution is less than 3.3 mL, the reduced SERS intensity at 1265 cm^−1^ (Figure 2a) confirms an insufficient applied amount of reducing agent, leading to incomplete gold ion reduction, uneven particle size distribution, and insufficient interparticle electrostatic repulsion of the generated gold nanoparticles [18]. These nanoparticles are prone to uncontrolled agglomeration. The LSPR effect of agglomerated gold nanoparticles is greatly weakened, and the number of active sites available for binding to ciprofloxacin molecules is reduced, ultimately leading to a significant decrease in the SERS signal. When the applied amount of trisodium citrate solution is 3.3 mL, the SERS signal intensity reaches the peak value. This is likely because the ratio of trisodium citrate to the gold precursor achieves an optimal balance at this time, ensuring a sufficient and uniform reduction reaction, and the generated gold nanoparticles have a uniform size, good dispersibility, and no obvious agglomeration [18]. At this point, the abundant surface active sites can provide sufficient adsorption anchors for ciprofloxacin molecules in chicken blood, while the strong LSPR field can efficiently amplify the Raman scattering signal, thereby maximizing the SERS intensity of ciprofloxacin in chicken blood. When the applied amount of trisodium citrate exceeds 3.3 mL, the SERS intensity of ciprofloxacin in chicken blood decreases with the increase in its applied amount. This may be attributed to the formation of a dense adsorption layer by excessive citrate ions on the surface of gold nanoparticles, which, on the one hand, occupies the active sites on the particle surface and hinders the effective adsorption of ciprofloxacin molecules onto the gold colloid surface; on the other hand, the excessive stabilizer leads to excessively strong electrostatic repulsion between particles, leading to the increase in the distance between gold nanoparticles, the weakening of the electromagnetic coupling effect among particles, and thus a subsequent decrease in the SERS enhancement effect [18].

The distinct changes in SERS intensity at 1265 cm^−1^ (Figure 2b) indicate that the difference in the SERS effect of these four electrolytes is related to ion type, ion valence, and their interaction with gold nanoparticles [19]. Na^+^ and Cl^−^ in NaCl synergistically neutralize the surface charge of gold nanoparticles, induce controllable aggregation to form high-density hot spots, and reduce the non-specific adsorption of macromolecules in chicken blood through the salting out effect, thus optimizing the contact sites for the target molecule, ciprofloxacin [19]. The SERS intensity of the blank control group without electrolyte reflects the background signal of ciprofloxacin in chicken blood under the condition of no electrolyte solution. At this time, the aggregation of gold colloid is only regulated by trace ions in chicken blood itself. High-valence ions such as Ca^2+^ and Mg^2+^ cause excessive aggregation of gold nanoparticles or non-specific wrapping due to excessive charge shielding or competition with biological macromolecules for adsorption sites, destroying the uniformity of hot spots and leading to signal attenuation [19]. K^+^ in KCl solution has a larger ionic radius and weaker hydration ability, resulting in insufficient precision in regulating aggregation, so it only slightly inhibits the SERS signal.

The variation trend of SERS intensity at 1265 cm^−1^ (Figure 2c) shows that the applied amount of gold colloid affects the SERS intensity by regulating the density of gold nanoparticles and the distribution of hot spots [20]. In this study, when the gold colloid applied amount was low (400 to 450 μL), the number of gold nanoparticles in the system was insufficient, and the number of SERS hot spots formed was limited, which could not effectively enhance the Raman signal of ciprofloxacin in chicken blood [21]. When the gold colloid applied amount increased to 500 μL, the gold nanoparticles were evenly distributed and the number of hot spots reached the peak value, and their interaction with ciprofloxacin molecules in chicken blood was the strongest, so the signal intensity was the highest [20]. However, when the gold colloid applied amount exceeded 500 μL, there were too many gold nanoparticles in the system, which were prone to excessive agglomeration, leading to the destruction of the hot spot structure between gold nanoparticles. At the same time, the competitive adsorption of macromolecules such as proteins in the chicken blood matrix increased, which instead reduced the effective adsorption efficiency of ciprofloxacin (the target molecule) in chicken blood, resulting in a decrease in SERS signal intensity [22]. Although the SERS signal intensity slightly rebounded at 600 μL, it was still lower than that at 500 μL. Therefore, 500 μL is the optimal applied amount of gold colloid, which can maximize the SERS signal intensity at the characteristic peak of 1265 cm^−1^ and effectively inhibit matrix interference.

In this study, an appropriate applied amount of NaCl solution (50 μL) leads to the maximum SERS intensity at the characteristic peak of 1265 cm^−1^ (Figure 2d), which is because this applied amount can neutralize the negative charge on the surface of gold nanoparticles, induce their moderate aggregation to form high-density hot spots, and avoid the non-specific adsorption of macromolecules such as proteins in chicken blood from occupying active sites, thus improving the SERS characteristic peak signal intensity of ciprofloxacin (the target molecule) in chicken blood [23]. When the applied amount of NaCl solution was too low (40 μL ≤ V < 50 μL), the aggregation was insufficient, the number of hot spots was small, and the SERS characteristic peak signal intensity was reduced. When the applied amount of NaCl solution was too high (>50 μL and ≤60 μL), excessive aggregation led to the formation of large-sized aggregates of gold nanoparticles, the uniformity of hot spots was destroyed, and Cl^−^ might compete with ciprofloxacin for adsorption sites on the surface of gold nanoparticles, further reducing the SERS characteristic peak signal intensity [23]. Therefore, 50 μL NaCl solution is the optimal applied amount for inducing controllable aggregation and optimizing the SERS signal.

At an adsorption time of 0 min, the ciprofloxacin molecules in chicken blood exhibited strong surface adsorption activity, and the gold nanoparticles had sufficient surface active sites. These molecules could be rapidly adsorbed onto the surface of gold nanoparticles after mixing with the gold colloid, with the adsorption amount reaching its maximum at this time, thereby yielding the strongest SERS signal. With the extension of adsorption time, components such as proteins in the test system would undergo competitive adsorption with ciprofloxacin molecules, occupying the active sites on the surface of gold nanoparticles, which led to a gradual decrease in the adsorption amount of ciprofloxacin and a subsequent decrease in SERS intensity [22]. When the adsorption time exceeded 2 min, the test system achieved adsorption equilibrium, and the adsorption amounts of ciprofloxacin molecules and impurity molecules tended to be stable, resulting in no significant change in SERS intensity thereafter [24]. Compared with other adsorption times, an adsorption time of 0 min can shorten the competitive adsorption time window of chicken blood matrix components, ensuring that ciprofloxacin occupies the active site of the gold colloid substrate before non-specific adsorption of matrix macromolecules and enhances SERS intensities at 1265 cm^−1^. Overall, an adsorption time of 0 min without additional incubation can maintain the maximum adsorption amount of the target molecule, ciprofloxacin, on the gold colloid surface and obtain the strongest SERS detection signal.

### 4.2. Spectral Preprocessing Analysis for Ciprofloxacin Detection in Chicken Blood

The above results demonstrate that the combination of air-PLS and SG smoothing can effectively remove high-frequency noise in Raman spectra and reduce noise interference during model fitting, thereby improving both calibration and prediction accuracy. In contrast, the combination of air-PLS and second-derivative preprocessing excessively amplified noise, resulting in decreased generalization ability of the GA-SVR model ($R_{p}^{2}$ value of 0.9315, RMSEP value of 3.4215). Although the $R_{p}^{2}$ value (0.9832) of the model using air-PLS plus first-derivative was higher than that using air-PLS alone, its $R_{c}^{2}$ value (0.9859) was slightly lower than that of air-PLS combined with SG.

The performances of the GA-SVR models using air-PLS combined with normalization, SNV, or MSC were not superior to those using air-PLS combined with SG smoothing. These methods failed to effectively improve model performance and even led to reduced predictive power in the present study. Among them, the RMSEP value (2.5242) of the air-PLS combined with the SNV model was higher than that of air-PLS alone; air-PLS combined with MSC caused excessive standardization, which seriously weakened the discrimination of effective signals ($R_{p}^{2}$ value of 0.9179, RMSEP value of 4.1333). Based on the comprehensive comparison, air-PLS combined with SG smoothing was determined as the optimal spectral preprocessing strategy for SERS-based quantitative detection of ciprofloxacin in chicken blood.

### 4.3. Analysis of GA Feature Extraction for Ciprofloxacin Detection in Chicken Blood

When the number of selected variables was small, RMSECV was relatively high, indicating that the model fitting ability was limited due to insufficient effective features. As key variables were gradually introduced, RMSECV declined sharply and model accuracy was significantly improved. When the number of variables increased to approximately 30 to 35, RMSECV decreased to a low level and remained stable. Further increasing the number of variables provided no obvious improvement in model accuracy, but instead increased model complexity and the risk of overfitting.

Combined with the optimized results of the F-test criterion, the number of SERS spectral variables was reduced from the original 450 to 33. This feature extraction based on GA not only simplifies the model but also maximizes the elimination of chicken blood matrix interference. The 33 selected characteristic Raman shifts are highly correlated with the concentration of ciprofloxacin in chicken blood, effectively filtering out redundant matrix information in the original SERS spectrum. This not only simplified the structure of the subsequent GA-SVR model but also improved its prediction accuracy and robustness, laying a solid foundation for constructing a high-performance quantitative detection model for ciprofloxacin in chicken blood.

### 4.4. Analysis of Quantitative Prediction Model for Ciprofloxacin Detection in Chicken Blood

A complex nonlinear relationship exists between the SERS spectra of chicken blood samples and ciprofloxacin concentration within 1 to 40 mg/L. The RBF kernel function possesses excellent nonlinear mapping capability, enabling it to effectively capture the nonlinear correlation between spectral data and analyte content, thus delivering superior fitting and prediction performance in the present system. Although the sigmoid kernel has nonlinear modeling potential, it tends to lose information from marginal samples due to its saturation characteristic, leading to relatively lower R_c_^2^ value (0.9805) and R_p_^2^ value (0.9784), as well as higher RMSEC value (1.6003) and RMSEP value (1.9832). The linear kernel function can only describe linear relationships and is insufficient to characterize the nonlinear features of the SERS spectral data in this study. Meanwhile, the polynomial kernel function is prone to overfitting with high-dimensional spectral data, resulting in reduced generalization ability. In summary, the RBF kernel function exhibits the best fitting accuracy and prediction reliability. Therefore, the RBF kernel function was selected for the GA-SVR prediction model in the subsequent SERS detection of ciprofloxacin in chicken blood. In the SERS spectra of chicken blood samples containing different concentrations of ciprofloxacin (Figure S5), the SERS intensities at 1265 cm^−1^ generally increased with increasing ciprofloxacin concentration, and exhibited a nonlinear relationship with the ciprofloxacin concentrations in chicken blood. Furthermore, ciprofloxacin exerted significantly different effects on the intensities of various SERS characteristic peaks in the chicken blood, with both the magnitude and trend of these intensity changes varying across different concentrations. By leveraging the characteristic peak at 1265 cm^−1^, this method could achieve the detection of ciprofloxacin in chicken blood with a minimum concentration of 1 mg/L.

The MLR model assumes a linear relationship between SERS spectral data and the content of the target analyte (ciprofloxacin). However, the actual nonlinear correlation between the SERS spectra of chicken blood samples and ciprofloxacin concentration limits its prediction accuracy, with relatively high RMSEC (1.5530) and RMSEP (2.5069) values. This also indicates that the MLR model cannot adequately characterize the complex nonlinearity of the chicken blood matrix or the high-dimensional redundant information of SERS spectra. Although the PLSR model can effectively handle the multicollinearity issue of spectral data, it performed poorly in the SERS detection of ciprofloxacin in chicken blood. Its R_c_^2^, R_p_^2^, RMSEC, and RMSEP values were also inferior to those of the other two models, reflecting that the PLSR model has limited capability to model the complex nonlinear data of the chicken blood matrix. In contrast, the GA-SVR model combines the feature extraction capability of the GA algorithm and the nonlinear fitting advantage of support vector regression. The GA algorithm is specifically used to screen for feature Raman shifts highly correlated with ciprofloxacin in chicken blood and insensitive to interference from the chicken blood matrix, and the SVR algorithm is used to fit the nonlinear relationship between the SERS spectrum of chicken blood and the concentration of ciprofloxacin in chicken blood. It accurately captures the nonlinear correlation between SERS spectral data and ciprofloxacin content through the RBF kernel function, while eliminating redundant variables, which effectively enhances the prediction accuracy and robustness of the model. Therefore, the GA-SVR model exhibited superior quantitative detection performance in this study, which is in good agreement with the previous optimization results of spectral preprocessing and kernel function selection.

## 5. Conclusions

This study developed a robust method combining SERS with GA-SVR for the rapid quantitative detection of ciprofloxacin in chicken blood. The combination of air-PLS and SG smoothing was validated as the optimal preprocessing strategy, which efficiently mitigated matrix-induced baseline drift and random noise. Subsequent feature extraction via the GA-F-test reduced the spectral variables from 450 to 33, thus simplifying the model architecture and improving its generalization ability.

Employing the RBF kernel function, the final GA-SVR model achieved excellent performance with a R_c_^2^ value of 0.9893, RMSEC value of 1.2953, R_p_^2^ value of 0.9874, and RMSEP value of 1.8617, outperforming MLR and PLSR models by accurately capturing the intrinsic nonlinear correlations in the complex chicken blood matrix. This SERS-GA-SVR method suitable for the detection of ciprofloxacin in chicken blood is not a simple combination of existing technologies, but a targeted improvement and integration of the chicken blood matrix, achieving rapid detection and minimal sample pretreatment. This SERS-GA-SVR approach enables sensitive, rapid detection with minimal sample pretreatment, providing a promising technical solution for veterinary drug residue monitoring in animal-derived foods. Future work will focus on expanding its applicability to multi-residue detection and real-world on-site supervision.

## Figures and Tables

**Table 1:** Results of GA-SVR models with different spectra pretreatment methods.

Pretreatment Method	$R_{c}^{2}$	RMSEC	$R_{p}^{2}$	RMSEP
air-PLS	0.9890	1.2183	0.9823	2.1567
air-PLS + SG	0.9893	1.2953	0.9874	1.8617
air-PLS + first derivative	0.9859	1.3885	0.9832	1.7080
air-PLS + second derivative	0.9812	1.5714	0.9315	3.4215
air-PLS + normalization	0.9886	1.2544	0.9793	2.0909
air-PLS + SNV	0.9891	1.2032	0.9575	2.5242
air-PLS + MSC	0.9886	1.4568	0.9179	4.1333

**Table 2:** Comparison of prediction performance for GA-SVR models with different types of SVR kernel functions.

Kernel Type	$R_{c}^{2}$	RMSEC	$R_{p}^{2}$	RMSEP
RBF	0.9893	1.2953	0.9874	1.8617
sigmoid	0.9805	1.6003	0.9784	1.9832
Linear	0.9797	1.6421	0.9769	2.1267
Polynomial	0.9843	1.4814	0.9697	2.3295

**Table 3:** Prediction performance comparison of different models.

Model	$R_{c}^{2}$	RMSEC	$R_{p}^{2}$	RMSEP
MLR	0.9885	1.5530	0.9512	2.5069
PLSR	0.9562	2.3948	0.9596	2.7370
SVR	0.9893	1.2953	0.9874	1.8617

## Data Availability

The original contributions presented in this study are included in the article. Further inquiries can be directed to the corresponding author.

## Supplementary Materials

The following supporting information can be downloaded at: https://www.mdpi.com/article/10.3390/bios16050259/s1, Figure S1: The overall experimental workflow of this study for rapid detection of ciprofloxacin residues in chicken blood. Figure S2: TEM images of seven gold colloids synthesized with different applied amounts of trisodium citrate solution: (a) 2.9 mL; (b) 3.1 mL; (c) 3.3 mL; (d) 3.5 mL; (e) 3.7 mL; (f) 3.9 mL; and (g) 4.1 mL. Figure S3: UV-vis spectra of seven gold colloids synthesized with different applied amounts of trisodium citrate solution. Figure S4: Comparison of spectral preprocessing effects for ciprofloxacin detection in chicken blood: (a) original spectrum; (b) air-PLS; (c) air-PLS + SG; (d) air-PLS + normalization; (e) air-PLS + first derivative; (f) air-PLS + second derivative; (g) air-PLS + SNV; and (h) air-PLS + MSC. Figure S5: Representative SERS spectra of chicken blood samples with different concentrations of ciprofloxacin.

## Abbreviations

The following abbreviations are used in this manuscript:

Air-PLS	adaptive iterative penalty least square method
SNV	standard normal variate
MSC	multiplicative scatter correction
$R_{c}^{2}$	coefficient of determination for the calibration set
$R_{p}^{2}$	coefficient of determination for the prediction set
RMSEC	root mean square error of calibration set
RMSEP	root mean square error of prediction set
SERS	surface-enhanced Raman spectroscopy
GA	genetic algorithm
SVR	support vector regression
HPLC	high-performance liquid chromatography
LC-MS/MS	liquid chromatography-tandem mass spectrometry
ELISA	enzyme-linked immunosorbent assay
SG	Savitzky–Golay
MLR	multivariate linear regression
PLSR	partial least squares regression
LSPR	local surface plasmon resonance
