# Supplementary material for: GA-SVR Optimized Surface-Enhanced Raman Spectroscopy for Rapid Detection of Ciprofloxacin Residues in Chicken Blood
Source: Biosensors (Basel). 2026 May 1;16(5):259. doi: 10.3390/bios16050259 (PMC13204726; doi:10.3390/bios16050259)
Supplement: Supplementary file 1 [file biosensors-16-00259-s001.zip › biosensors-4227961-supplementary.pdf]

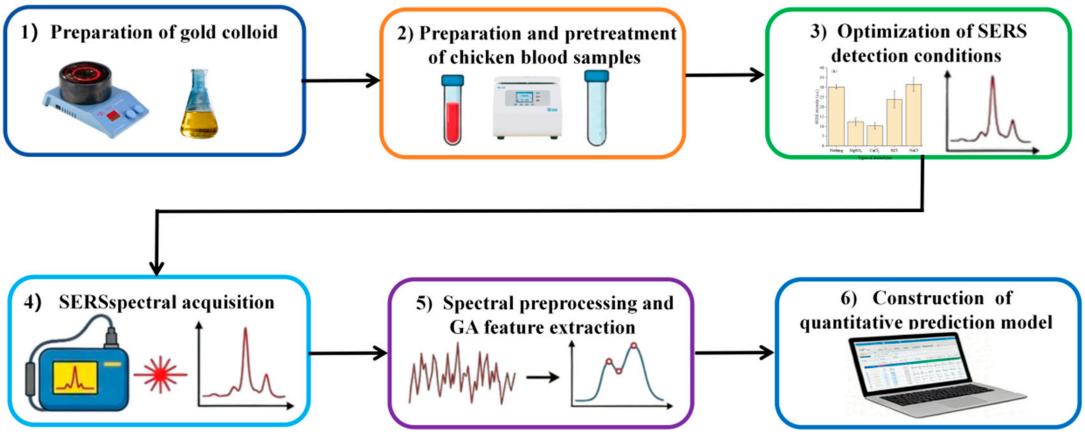

Figure S1 The overall experimental workflow of this study for rapid detection of ciprofloxacin residues in chicken blood.

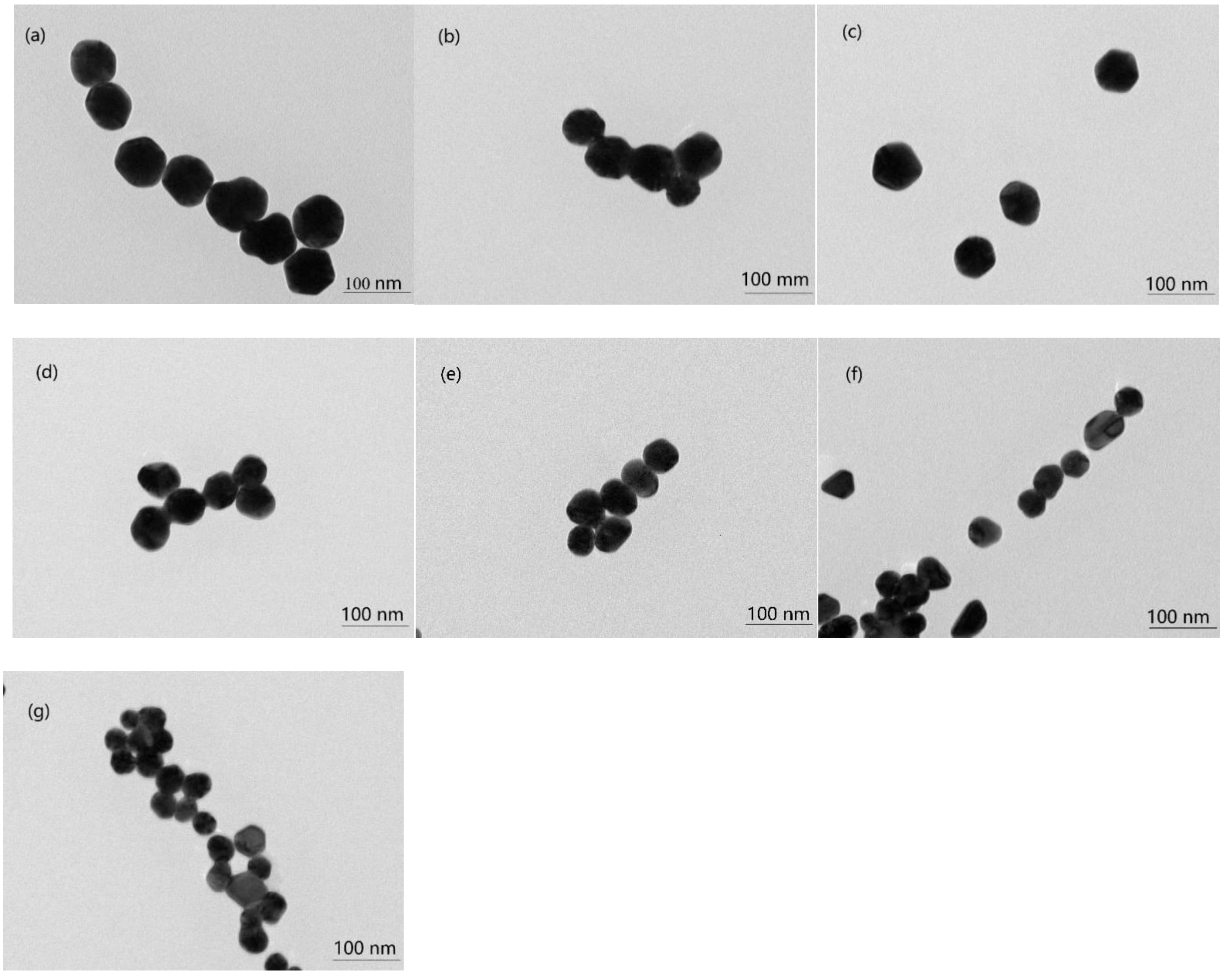

Figure S2 TEM images of seven gold colloids synthesized with different applied amounts of trisodium citrate solution: (a) 2.9 mL; (b)3.1 mL; (c)3.3 mL; (d) 3.5 mL; (e) 3.7 mL; (f) 3.9 mL; and (g) 4.1 mL.

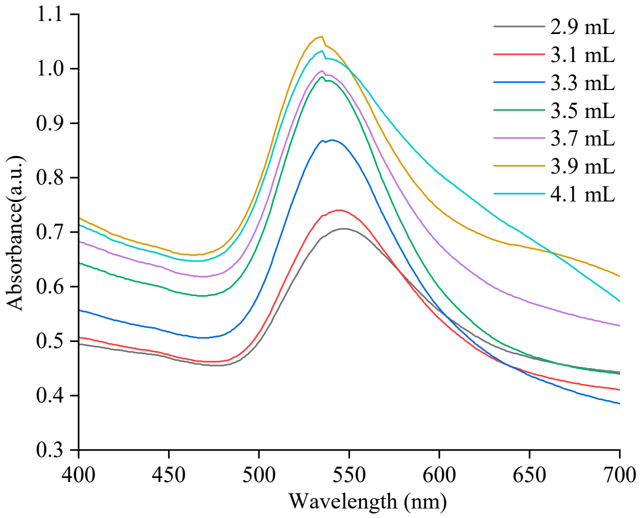

Figure S3 UV-vis spectra of seven gold colloids synthesized with different applied amounts of trisodium citrate solution.

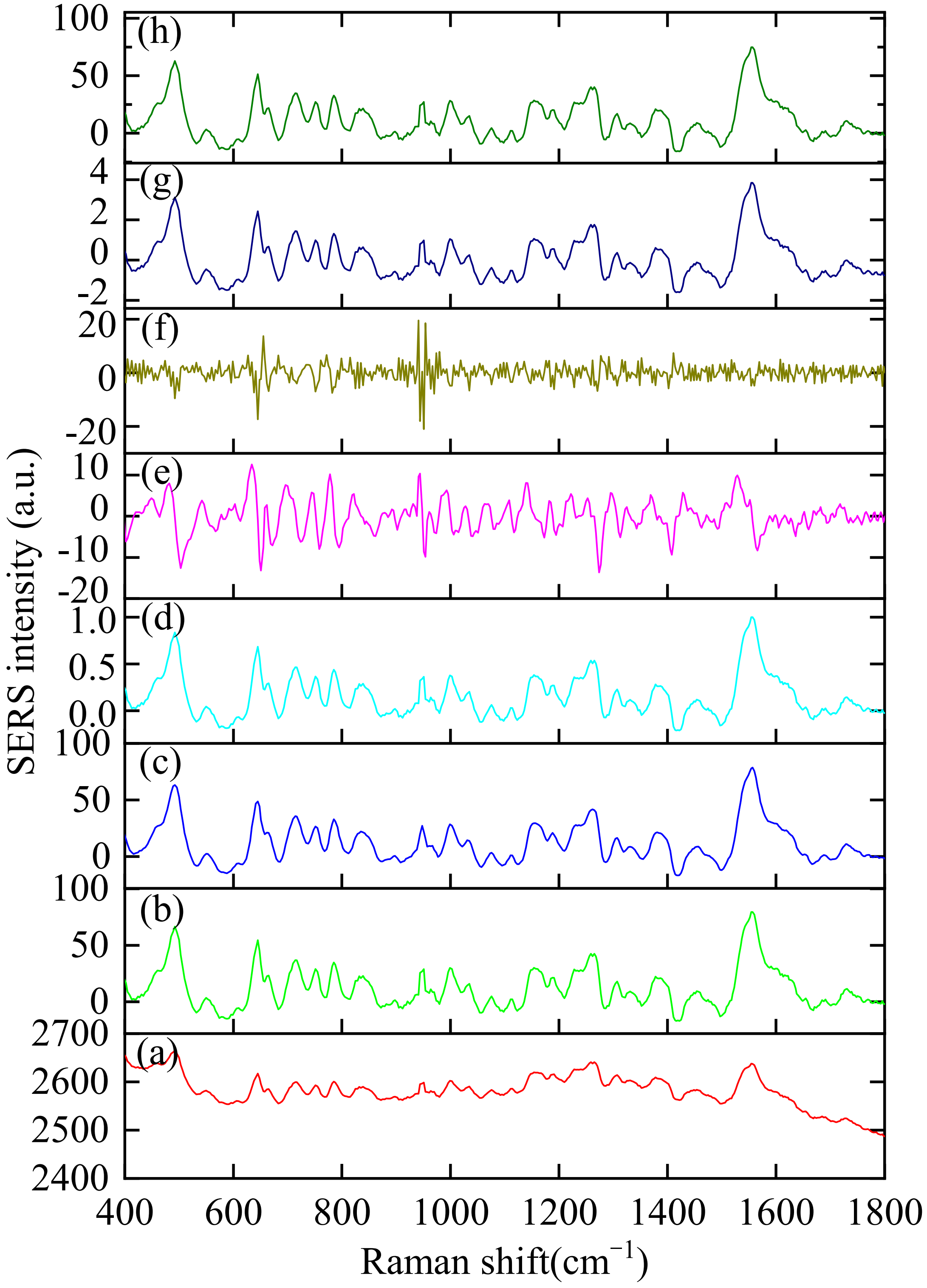

Figure S4 Comparison of spectral preprocessing effects for ciprofloxacin detection in chicken blood: （a） original spectrum； （b）air-PLS; （c）air-PLS+SG; （d）air-PLS+normalization; （e）air-PLS+first derivative; （f）air-PLS+second derivative; （g）air-PLS+SNV; and （h）air-PLS+MSC.

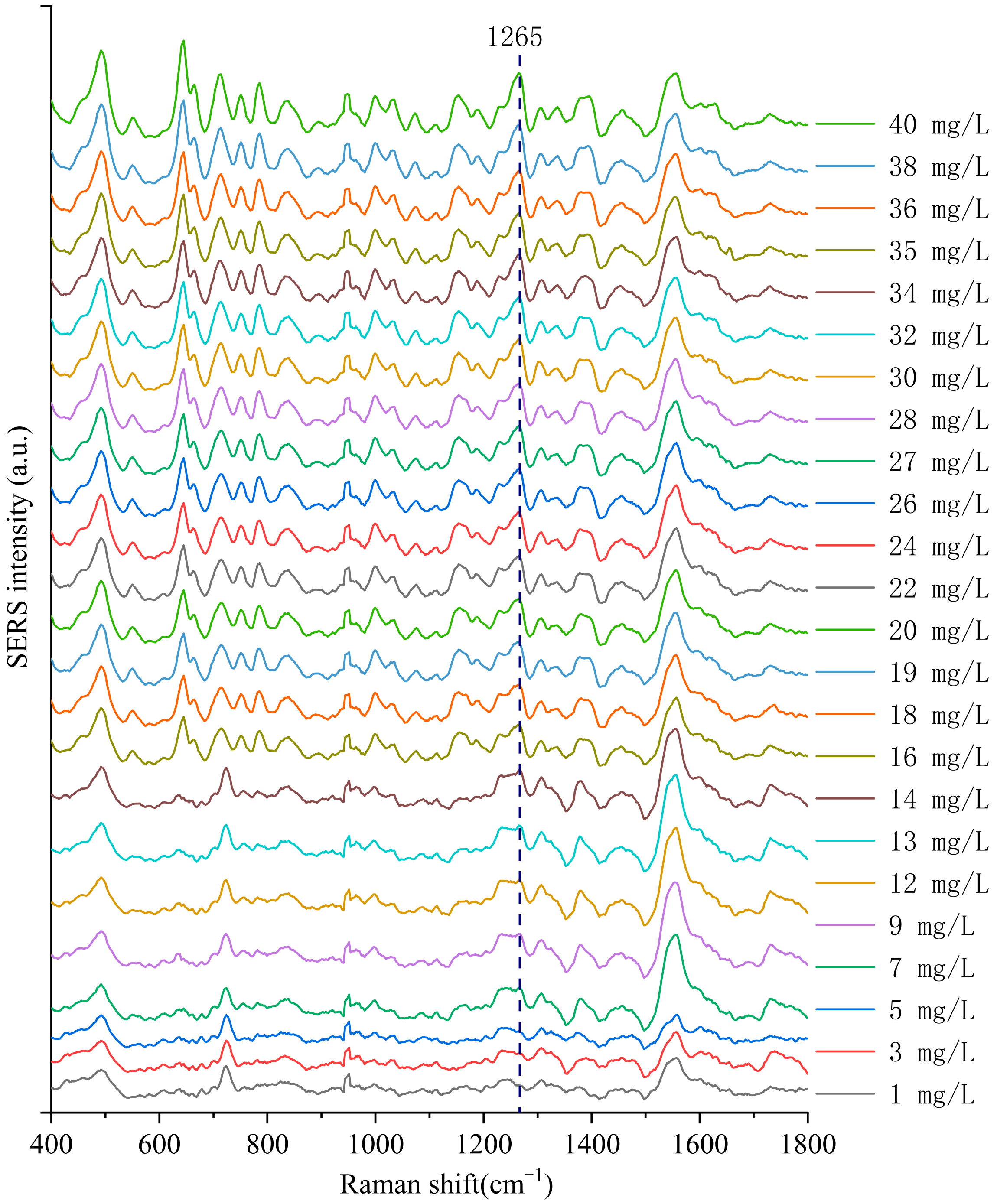

Figure S5 Representative SERS spectra of chicken blood samples with different concentrations of ciprofloxacin.
